# Case Report: Conservative management of hemophagocytic lymphohistiocytosis with fulminant hepatic failure in a pediatric leukemia patient

**DOI:** 10.3389/fped.2025.1719708

**Published:** 2025-11-13

**Authors:** Filipa Paixão, André Salvada, Susana Santos, Catarina Amorim, Carlos Escobar

**Affiliations:** 1 Pediatrics Department, Professor Doutor Fernando Fonseca Hospital, Lisbon, Portugal; 2 Pediatrics Department, Francisco Gentil Portuguese Institute of Oncology, Lisbon, Portugal

**Keywords:** hemophagocytic lymphohistiocytosis, acute liver failure, acute lymphoblastic leukemia, varicella zoster virus infection, immunosuppressive agents

## Abstract

**Introduction:**

Hemophagocytic lymphohistiocytosis (HLH) is a life-threatening hyperinflammatory syndrome that can lead to multiorgan failure, including acute liver failure. While varicella infection in leukemia patients in remission is not uncommon, HLH complicated by fulminant hepatic failure is exceedingly rare.

**Case report:**

We report the case of an adolescent with acute T-cell lymphoblastic leukemia in remission who developed varicella infection, followed by decompensated shock and acute liver failure (ALF). The patient met HLH-2004 diagnostic criteria, including fever, pancytopenia, hypertriglyceridemia, hyperferritinemia, elevated soluble interleukin-2 receptor, and bone marrow hemophagocytosis. The patient received immunosuppressive therapy according to the HLH-2004 protocol, along with antiviral therapy and supportive care, and achieved full recovery without liver transplantation. To our knowledge, this is among the few reported cases of HLH-associated acute liver failure in a pediatric leukemia patient successfully managed conservatively.

**Discussion:**

This case highlights the diagnostic challenges of HLH in immunocompromised children, where early signs such as fever and cytopenias may be misattributed to sepsis or chemotherapy-related complications. It underscores the importance of including HLH in the differential diagnosis of ALF in high-risk patients and demonstrates that prompt recognition and targeted treatment of the hyperinflammatory state can be lifesaving, even in severe presentations.

## Introduction

Hemophagocytic lymphohistiocytosis (HLH) is a hyperinflammatory syndrome marked by dysregulated immune activation, potentially leading to life-threatening multi-organ failure. HLH can be primary (due to genetic mutations), or secondary, triggered by infections (e.g., Epstein–Barr Virus, Cytomegalovirus, Adenovirus), malignancy or autoimmune conditions. It involves excessive activation of T cells, natural killer cells and macrophages, with uncontrolled cytokine release (1).

According to the HLH-2004 diagnostic criteria, a diagnosis can be made either by identifying a known genetic mutation associated with HLH or by fulfilling five of eight criteria (Table 1) (2).

**Table 1:** Diagnostic criteria for hemophagocytic lymphohistiocytosis (2).

Number	Diagnostic criteria
1	Fever
2	Splenomegaly
3	Cytopenia in ≥2 cell lineages: - Hemoglobin <9 g/dL, in neonates <10 g/dL - Platelet count <100 × 10^3^/mL; neutrophil count <1 × 10^3^/mL
4	Hypertriglyceridemia (>265 mg/dL) or hypofibrinogenemia (<150 mg/dL)
5	Hyperferritinemia (>500 ng/mL)
6	Soluble IL-2 receptor (sCD25) >2,400 U/mL (or above the normal limits established by the laboratory)
7	Hemophagocytosis in bone marrow, spleen, lymph nodes or liver
8	Low or absent NK-cell activity

The primary treatment goal is to suppress the excessive inflammatory response. The HLH-1994 and HLH-2004 studies, conducted by the Histiocyte Society, led to the development of a treatment protocol that remains the standard of care today. This regimen includes etoposide and dexamethasone, which have significantly improved survival (1).

Acute liver failure (ALF) is a rare but often fatal complication of HLH, with limited guidance available for its management. To the best of our knowledge, there appear to be only two reports of patients with HLH and acute liver failure, successfully treated without hepatic transplantation (3, 4).

## Case report description

This case describes a 16-year-old male with autism spectrum disorder and acute T-cell lymphoblastic leukemia, undergoing treatment with the “All Together” chemotherapy protocol (5). He was in the delayed intensification stage at the time of presentation. He was admitted with a 24 h history of fever. Initial bloodwork revealed hemoglobin of 9.4 g/dL, leukopenia (660/μL) with neutropenia (190/μl), platelets 198 × 10^3^/µL, and a C-reactive protein (CRP) of 0.23 mg/dL. Given fever and neutropenia in a patient in leukemia remission, empirical antibiotic therapy with amikacin (15 mg/kg/day, once daily) and piperacillin/tazobactam (75 mg/kg/dose, every 8 h) was initiated.

On the third day of hospitalization, the patient developed a vesicular rash suggestive of varicella infection. He had no prior history of varicella. His mother reported his brother had varicella two weeks earlier. Acyclovir (20 mg/kg/dose, every 8 h) and vancomycin (40 mg/kg/day, every 6 h) were added, and the patient was transferred to another hospital ward to limit contagion. On the sixth day, the patient developed persistent hypotension (systolic 75 mmHg, diastolic 37 mmHg, heart rate 160 bpm), with a peak lactate level of 5.6 mmol/L and hemoglobin of 6.6 g/dL. Hemodynamic stabilization was achieved with a bolus of normal saline (10 mL/kg) and a transfusion of packed red blood cells (10 mL/kg). Due to continued clinical deterioration, he was transferred to the pediatric intensive care unit (PICU).

Upon PICU admission, he exhibited worsening hemodynamic instability requiring intravenous norepinephrine infusion (maximum dose 0.8 mcg/kg/min). Inflammatory markers increased (CRP 10.5 mg/dL, procalcitonin 6.5 ng/mL), and hematologic parameters further declined (hemoglobin 7.9 g/dL and platelets 55 × 10^3^/µL). He showed signs of hepatic injury (AST 2,419 U/L, ALT 887 U/L), cholestasis (total bilirubin 4.55 mg/dL, direct 3.9 mg/dL), and worsening liver function (INR 2.13-peak value, APTT 80.7 s, fibrinogen 0.6 g/L, albumin 1.52 g/dL). He also developed hypoglycemia (minimum 22 mg/dL) and hyperammonemia (119.6 μmol/L). Abdominal ultrasound revealed hepatomegaly (16 cm in greatest longitudinal axis), with regular contours and homogeneous echotexture. No abnormalities were observed in the spleen, gallbladder, or biliary tract.

Twenty-four hours after PICU admission, due to persistent pancytopenia (not consistent with remission-phase leukemia), ongoing fever, and evolving acute liver failure without an alternative etiology, HLH was suspected. Laboratory findings revealed markedly elevated ferritin (92,979 ng/mL), triglycerides (767 mg/dL), and subsequently soluble IL-2 receptor (sCD25) at 375 U/mL (laboratory reference range: 19–68 U/mL). All serological tests for hepatitis A, B, and C viruses, Epstein–Barr virus, and cytomegalovirus were negative, as were serial blood cultures. Bone marrow aspiration, performed 24 h after PICU admission, confirmed remission of leukemia and showed evidence of hemophagocytosis, supporting a diagnosis of HLH. Varicella-zoster virus DNA was detected in the blood via PCR (viral load 7,105 UI/mL), confirming active infection. After multidisciplinary discussion with pediatric oncology and infectious disease teams, treatment according to the HLH-2004 protocol was initiated.

The patient required hemodynamic support with norepinephrine until day 8. On day 9 of inpatient care the patient developed hypertension (systolic 160 mmHg) in likely relation to the hypertensive effects of dexamethasone, so he initially started sublingual nifedipine 10 mg every four hours and around D14 transitioned to captopril 25 mg every 8 h, which was stopped as he was weaned off dexamethasone and achieved normotension.

Hematologic support included one dose of vitamin K (10 mg), four transfusions of packed red blood cells, 8 transfusions of platelets, 4 units of plasma, and 5 units of fibrinogen concentrate. Under the HLH-2004 protocol, recovery of hemoglobin, neutrophil, and platelet counts was delayed, likely due to the bone marrow-suppressive effects of etoposide. Ferritin levels gradually decreased over time.

Hepatic and gastrointestinal function improved steadily under HLH-directed therapy. The INR normalized within 48 h of initiating the HLH-2004 protocol. In contrast, normalization of cytolysis and cholestasis parameters occurred more gradually. Lactulose was not administered, as there were no clinical signs of hepatic encephalopathy.

On day 18 of hospitalization, the patient was transferred to the oncology unit in stable condition, where a lumbar puncture was performed for cerebrospinal fluid analysis, which was negative for central nervous system involvement by HLH.

In total, he completed 21 days of acyclovir and eight weeks of treatment according to the HLH-2004 protocol, which included four weeks of etoposide at a dose of 150 mg/m^2^ (administered twice weekly during the first two weeks and once weekly thereafter until week four). Dexamethasone was administered for eight weeks, starting at 10 mg/m^2^ daily for the first two weeks and tapered over the following six weeks. No dose adjustments were required. Given the patient's favorable response to the treatment protocol, no additional therapeutic interventions were deemed necessary. After completing the protocol, a repeat bone marrow examination showed no evidence of hemophagocytosis. Four months after discharge he remains clinically stable.

**Figure 1:**
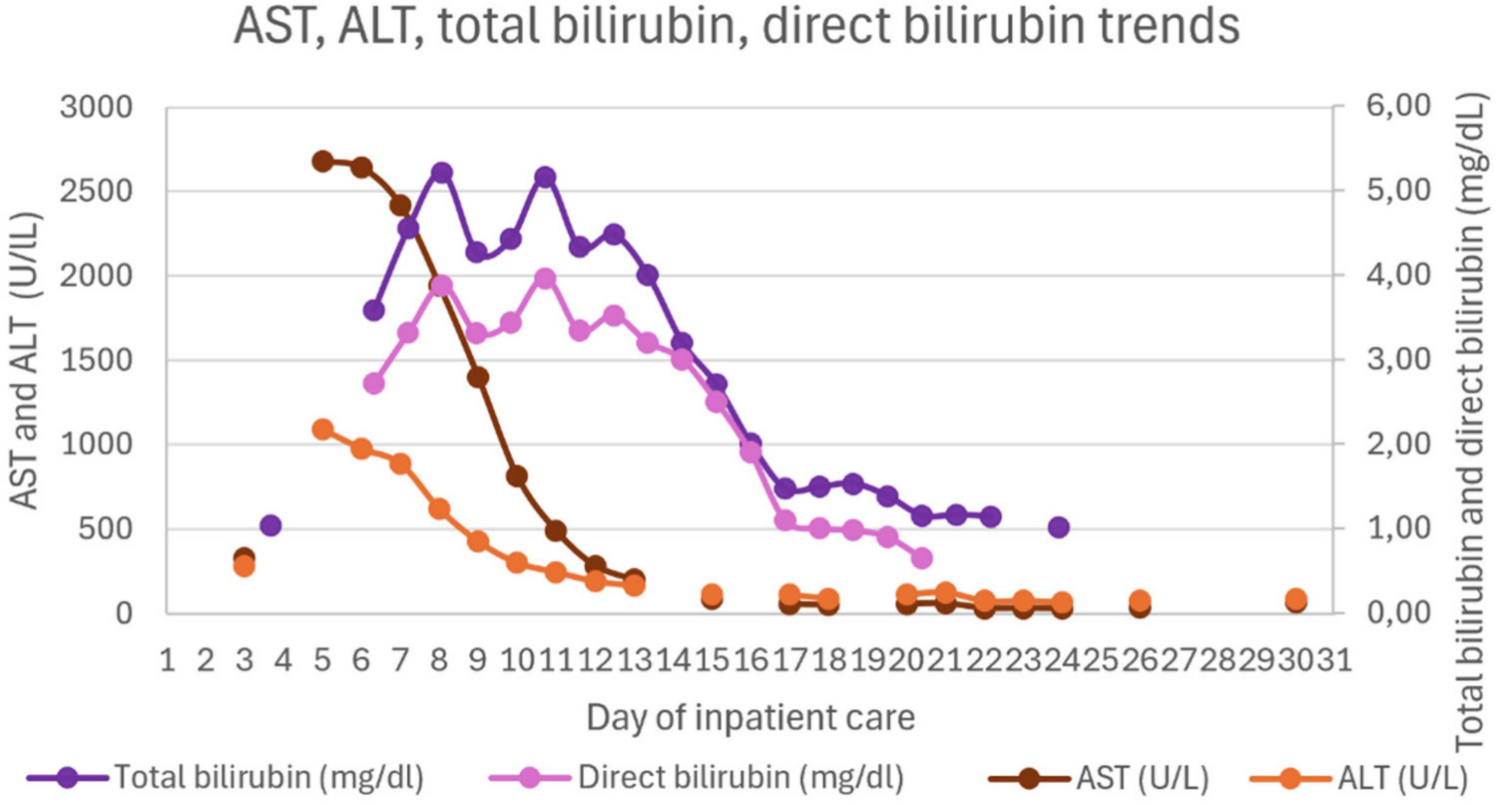
AST, ALT, total bilirubin and direct bilirubin trends.

**Figure 2:**
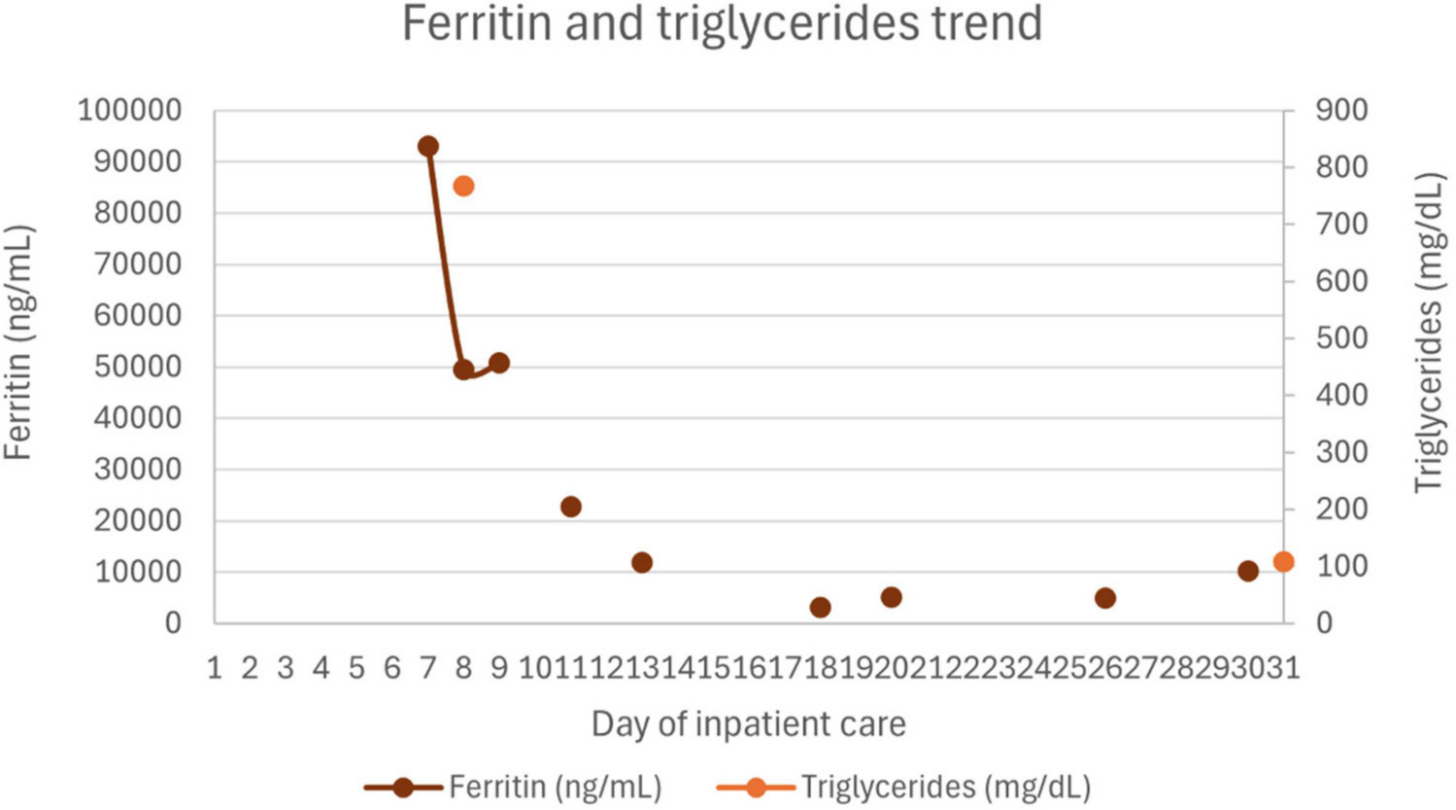
Ferritin and triglycerides trends.

**Figure 3:**
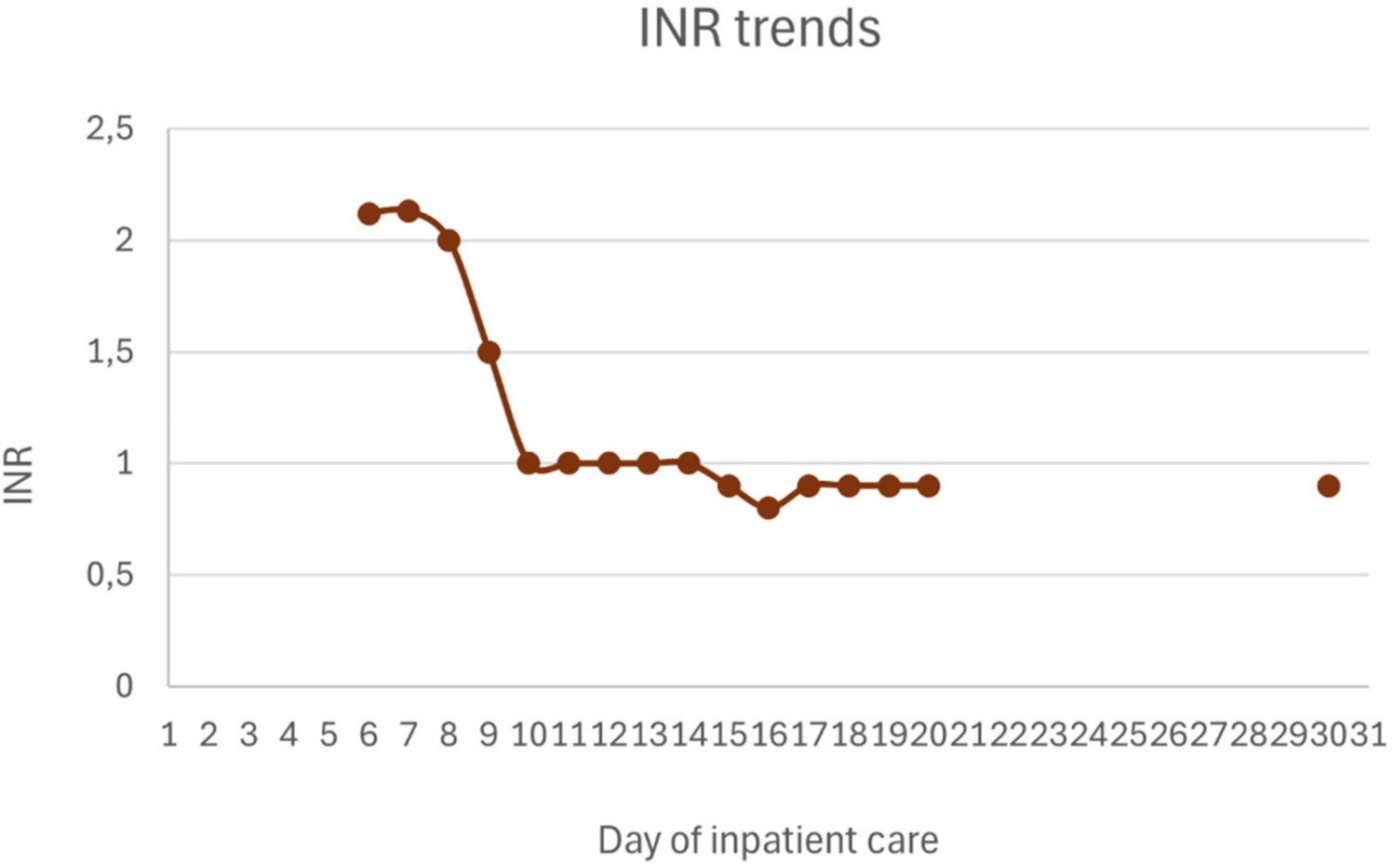
INR trends.

**Figure 4:**
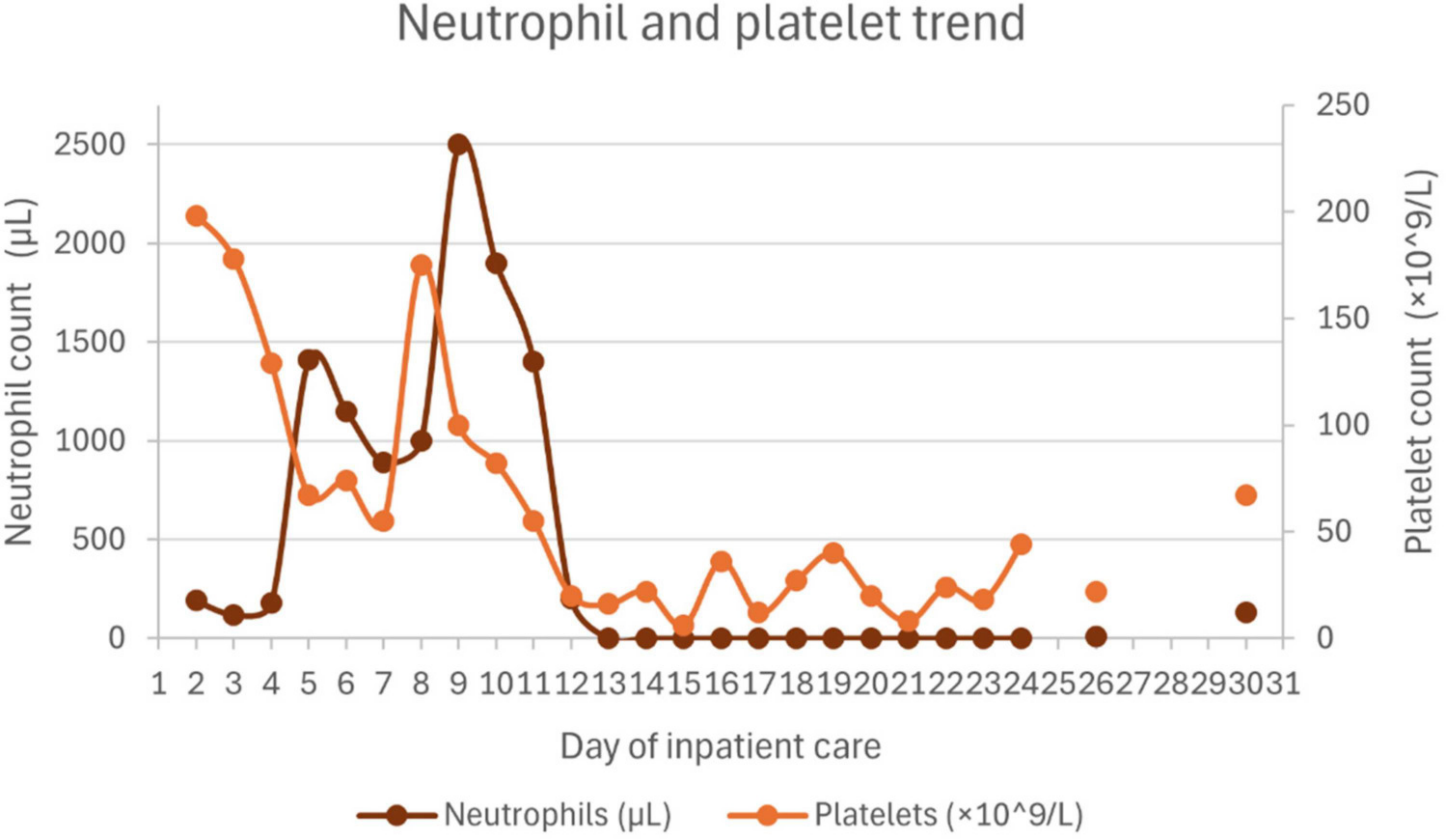
Neutrophil and platelet count trends.

**Figure 5:**
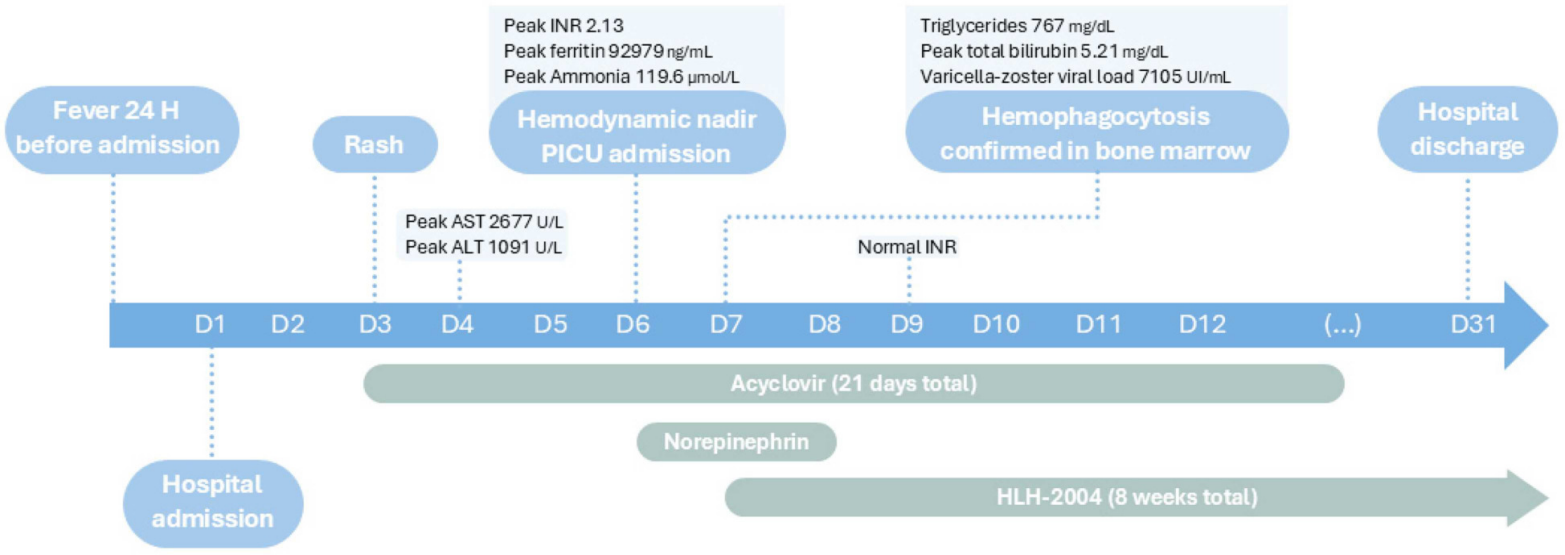
Timeline of events during inpatient care.

Figures 1–4 illustrate trends in blood analyses and Figure 5 provides a timeline of events for visual reference.

## Discussion

HLH is a potentially fatal condition due to the risk of acute multiorgan failure. We report the case of an adolescent with leukemia in remission who developed varicella infection, which is not uncommon in such patients. However, his infection was unusually complicated by HLH and ALF, and, remarkably, he recovered without requiring liver transplantation. To the best of our knowledge, this is among the very few reported cases of HLH-associated acute liver failure treated successfully without transplantation. We highlight two similar cases: a neonate with HSV-1 (2008) and a 16-year-old with varicella (2018). Both patients were managed with antiviral therapy and immunosuppressive treatment—methylprednisolone and intravenous immunoglobulin in the first case, and etoposide plus dexamethasone in the second (3). Our case also represents one of the very few reported cases of HLH complicated by ALF in a leukemia patient successfully treated without transplantation. Liver transplantation is reserved for patients unresponsive to medical therapy, but mortality remains high due to surgical complications and long-term immunosuppression (3, 6).

The clinical diagnosis of HLH remains challenging, particularly in immunocompromised patients where signs such as fever or cytopenia may initially be attributed to sepsis or chemotherapy-related complications. Differentiating HLH from sepsis can be challenging. Key distinguishing features of HLH include persistent fever, significant cytopenias requiring multiple transfusions, and hypofibrinogenemia, although the latter may also be observed in sepsis complicated by disseminated intravascular coagulation. Hepatosplenomegaly is more commonly associated with HLH than sepsis, providing an important diagnostic clue. The presence of hemophagocytosis in bone marrow examination supports the diagnosis of HLH, however, critically ill patients without HLH may also demonstrate hemophagocytosis in the absence of other diagnostic criteria (7).

Patients with active malignancies, particularly lymphoma, may present with an HLH-like syndrome driven directly by the underlying malignancy. In these cases, antineoplastic therapy should take priority over HLH-directed therapies, although these therapeutic approaches commonly overlap (7). In patients with malignancy receiving immunosuppressive treatment who develop HLH secondary to infection, as in our case, the underlying pathophysiology is not fully understood. It is thought to result from a dysregulated immune response to infection in the setting of immunosuppression. These patients are thought to respond poorly to treatment, although corticosteroids and etoposide are commonly used (7). However, our patient exhibited a favorable response to this therapeutic approach.

This clinical case also highlights broader questions about immune homeostasis in patients with leukemia, in whom altered immune responses may predispose to immune-mediated complications. A 2021 multivariate analysis showed that certain subgroups of pediatric leukemia patients following HSCT may be at increased risk of downstream effects, including relapse, depending on T- and NK-cell reconstitution dynamics (8). Further studies are needed to clarify the mechanisms of immune dysregulation in leukemia patients who develop HLH secondary to infection in the context of immunosuppression.

In our case, early suspicion followed by prompt initiation of both varicella- and HLH-directed therapy, contributed to a favorable outcome. This case highlights the importance of considering HLH in the differential diagnosis of acute liver failure, particularly when accompanied by cytopenia and systemic inflammation in a high-risk patient. Despite the severity of hepatic involvement, liver function normalized progressively with supportive care and immunosuppressive therapy, without the need for liver transplantation.

Our case suggests that even in HLH-associated ALF, conservative management may suffice when the underlying hyperinflammatory state is effectively controlled. Early identification of the underlying cause of HLH, along with prompt initiation of targeted therapy, immunosuppression, and supportive care, is crucial for managing this potentially fatal condition (3).

## Data Availability

The original contributions presented in the study are included in the article/Supplementary Material, further inquiries can be directed to the corresponding author.
